# High rhesus (Rh(D)) negative frequency and ethnic-group based ABO blood group distribution in Ethiopia

**DOI:** 10.1186/s13104-017-2644-3

**Published:** 2017-07-26

**Authors:** Lemu Golassa, Arega Tsegaye, Berhanu Erko, Hassen Mamo

**Affiliations:** 1 Aklilu Lemma Institute of Pathobiology, Addis Ababa University, P.O. Box 1176, Addis Ababa, Ethiopia; 2 Jimma University, P.O. Box 378, Jimma, Ethiopia; 3 Department of Microbial, Cellular and Molecular Biology, College of Natural Sciences, Addis Ababa University, P.O. Box 1176, Addis Ababa, Ethiopia

**Keywords:** ABO-Rh(D), Blood group, ‘Highlanders’, Nilotics, Gambella, Ethiopia

## Abstract

**Background:**

Knowledge of the distribution of ABO-Rh(D) blood groups in a locality is vital for safe blood services. However, the distribution of these blood systems among Ethiopians in general is little explored. This study was, therefore, designed to determine the ABO-Rh(D) blood group distribution among patients attending Gambella hospital, southwestern Ethiopia.

**Methods:**

A cross-sectional study was conducted between November and December 2013 (N = 449). The patients were grouped into two broad categories. Those who originally moved from different parts of Ethiopia and currently residing in Gambella are named ‘highlanders’ (n = 211). The other group consisted of natives (Nilotics) to the locality (n = 238). ABO-Rh(D) blood groups were typed by agglutination, open-slide test method, using commercial antisera (Biotech laboratories Ltd, Ipswich, Suffolk, UK).

**Results:**

Overall, majority of the participants (41.20%) had blood type ‘O’ followed by types ‘A’ (34.96%), ‘B’ (20.48%) and ‘AB’ (3.34%). However, blood type ‘A’ was the most frequent (44.07%) blood group among the ‘highlanders’ and 50.42% of Nilotic natives had type ‘O’. The proportion of participants devoid of the Rh factor was 19.37%.

**Conclusions:**

While the ABO blood group distribution is similar to previous reports, the Rh(D) frequency is much higher than what was reported so far for Ethiopia and continental Africa.

## Background

Currently there are about 700 human blood group antigens organized into 35 systems [1]. Among these, the ABO and Rhesus (Rh(D)) are the most important systems. The ABO and Rh blood group antigens are investigated for understanding human inheritance and migration patterns. These blood group systems have also got vital clinical and practical significance. They are routinely screened in transfusion and transplantation, pregnancy, forensics, paternity testing and legal medicine [2]. Knowledge of distribution of ABO-Rh blood group is helpful for effective management of blood banks and safe blood transfusion services. Particularly, identification of the Rh system is important to avoid a potential risk of erythroblastosis fetalis.

The distribution of the ABO-Rh blood group varies markedly in different races and ethnic groups in different parts of the world [3]. Apart from the spatial and ethnic/racial variations, the ABO-Rh blood group frequencies may change temporally in a single population [4]. Variation of ABO gene distribution with socio-economic status was also demonstrated in England [5]. It is, therefore, imperative to have a reliable and up-to-date data on the distribution of these blood groups in various populations.

Further, the ABO blood system is widely being studied in relation to susceptibility to infectious as well as non-infectious diseases in different human populations. The ABO blood group system is known to be associated with diverse forms of cancer such as that of skin [6], pancreace [7, 8], epithelial ovarian [9, 10] and gastric cancer [11], and other non-communicable diseases like ischaemic heart disease [12] and diabetes mellitus [13]. Among infectious diseases, the association of malaria with the ABO blood group is increasingly being recognized [14–16] following the initial suggestion 48 years back [14].

In Ethiopia, the distribution of the ABO-Rh blood group system is little explored. Thus, in this study the frequency distribution of these blood groups was determined among patients attending Gambella hospital, southwestern Ethiopia.

## Methods

The study was conducted at Gambella hospital in Gambella Town, southwestern Ethiopia, about 777 km from Addis Ababa. *Nuer* and *Anuak* are the largest native ethnic groups in Gambella and settlers of various ethnic origins who moved from all over Ethiopia are considered as ‘highlanders’. Consenting patients (for minors parental consent was obtained) visiting the hospital between November and December 2013 were included in the survey. Following an interview to capture socio-demographic parameters blood samples were drawn. ABO-Rh blood groups were typed on the spot by the open-slide agglutination method using commercial antisera (Biotech Laboratories Ltd, Ipswich, Suffolk, UK) as per the manufacturer’s instruction.

Data entered into Microsoft Excel spreadsheet, checked for correctness and analyzed. Blood group phenotypic frequencies were expressed in percentage and allele frequencies estimated under the assumption of Hardy–Weinberg equilibrium. The Chi-squared test was used to test the association between blood group and ethnic origin, as well as to compare observed allelic and genotypic frequency distributions of the blood groups. The level of statistical significance was at p<0.05.

## Results

Totally 449 participants (211 ‘highlanders’, 238 Nilotics) were included in the study, 210 males and 239 females. The mean age was 16 years, median 15 and range 1–90 years.

### ABO-Rh(D) blood group phenotype

Majority of the participants (41.20%) had blood type ‘O’ followed by type ‘A’ (39.96%) and ‘B’ (20.48%). Type ‘AB’ was the least frequent (3.34%). The result shows that the proportion of individuals having blood type ‘O’ or ‘A’ was significantly higher compared to those having type ‘B’ or AB (p<0.0001). The overall proportion of patients devoid of the Rh(D) was 19.37%. While blood type ‘A’ was the most frequent blood group among ‘highlanders’ (44.07%), over 50.42% of Nilotic natives had type ‘O’ (Table 1).

**Table 1:** Distribution (%) by population group of ABO and Rh(D) phenotypes in Gambella hospital, southwestern Ethiopia

Population	Number	ABO phenotypes				Rh(D) phenotypes	
		A	B	AB	O	Rh(D)+	Rh(D)−
Nilotics	238	26.89	20.58	2.10	50.42	82.35	17.64
‘Highlanders’	211	44.07	20.37	4.73	30.80	78.67	21.32
Total	449	34.96	20.48	3.34	41.20	80.62	19.37

The frequency of blood group ‘O’ among the natives was significantly higher compared to that among ‘highlanders’ (30.8%) with a p-value of <0.0001. The frequency distribution of the Rh(D) within the ABO system is indicated in Table 2 with the highest Rh negativity frequency occurring in blood group ‘O’.

**Table 2:** Distribution (%) of Rh(D) phenotypes among ABO blood group carriers in Gambella hospital, southwestern Ethiopia

Population	Rh(D)	ABO				Total
		A	B	AB	O	
Nilotics	Positive	22.68	15.54	2.10	42.01	82.35
	Negative	4.20	5.04	0.00	8.40	17.64
‘Highlanders’	Positive	36.01	16.11	4.73	22.27	78.67
	Negative	8.05	4.26	0.47	8.53	21.32
Total	Positive	28.90	15.84	3.10	32.70	80.62
	Negative	6.00	4.60	0.20	8.40	19.37

### ABO-Rd(D) allelic frequencies

Overall, allelic frequencies of O, A and B were 0.6418, 0.2305 and 0.1277 respectively. While the frequency of allele O among the Nilotics was 0.7100, for the ‘highlanders’ it was 0.5549. The frequencies of D and d alleles among the Nilotics were 0.5800 and 0.4200 respectively. Among ‘highlanders’ the allelic frequency of D was 0.5382, and that of d was 0.4617 (Table 3).

**Table 3:** Gene frequencies of ABO and Rh(D) blood groups alleles among patients in Gambella hospital, southwestern Ethiopia

Population	Gene (allele)	Frequency	Genotype	Frequency	Phenotype	Frequency (%)
Nilotics	O(r)	0.7100	OO	0.5041	O	50.41
	A(p)	0.1697	AA	0.0287	A	2.87
	B(q)	0.1203	AO	0.2409	A	24.09
			BB	0.0144	B	1.44
			BO	0.0854	B	8.54
			AB	0.0289	AB	2.89
	D	0.5800	DD	0.3364	Rh(D)+ve	33.64
			Dd	0.4872	Rh(D)+ve	48.72
	d	0.4200	dd	0.1764	Rh(D)−ve	17.64
‘Highlanders’	O(r)	0.5549	OO	0.3079	O	30.79
	A(p)	0.3103	AA	0.0962	A	9.62
	B(q)	0.1348	AO	0.3443	A	34.43
			BB	0.0181	B	1.81
			BO	0.1496	B	14.96
			AB	0.0836	AB	8.36
	D	0.5382	DD	0.2896	Rh(D)+ve	28.96
			Dd	0.4969	Rh(D)+ve	49.69
	d	0.4617	dd	0.2131	Rh(D)−ve	21.31

The observed and expected frequencies of the ABO blood groups were not significantly different in both population groups (Table 4).

**Table 4:** Observed and expected frequencies of the ABO blood group among the Nilotics and ‘highlanders’ in Gambella hospital, southwestern Ethiopia

Population	ABO group	Observed(o)	Expected(e)	Deviation(d)	d^2^/e
Nilotics	A	64	64.2070	−0.2070	0.0006
	B	49	44.0992	4.9008	0.5460
	AB	5	9.7175	−4.7175	2.2901
	O	120	119.9758	0.0242	0.0001
X^2^ (∑d^2^/e)					2.8368
‘Highlanders’	A	93	92.9786	0.0214	0.0000
	B	43	35.3999	7.6001	1.6316
	AB	10	17.6516	−7.6516	3.3168
	O	65	64.9698	0.0302	0.0000
X^2^ (∑d^2^/e)					4.9484

## Discussion

In this study, blood type ‘O’ was the most dominant types followed by ‘A’, ‘B’ and ‘AB’. Nationwide ABO-Rh(D) blood group data is lacking in Ethiopia. Mason and colleagues, perhaps in a first attempt, screened 878 Ethiopian soldiers who were participating in the Korean War in the late 1940s and found ‘O’, ‘A’, ‘B’ and ‘AB’ to be 41.2, 28.5, 24.0 and 6.3%, respectively [17]. In the 1960s it was reported that type ‘A’ frequencies were about 20% and ‘B’ 16% in Tigre, Amhara and other population groups in the country [18].

Probably the only published study from Ethiopia that used relatively large sample size (164,380) was that of Seifu and Dagne some 30 years back [19]. That study extracted several years hospital and red cross data and found the frequencies of ‘O’, ‘A’, ‘B’ and ‘AB’ to vary from 40–54, 25–31, 17–25 and 2–8% respectively. The donors were from northern, western and central parts of the country including Addis Ababa, the capital. Later fragmented and small-scale studies from different parts of the country reported comparable results [20–22].

In this study, while type ‘O’ was dominant overall and among the Nilotic natives of Gambella, type ‘A’ had a significantly higher frequency among the ‘highlanders’ who belong to different ethnic groups. Blood type ‘O’ is 50% and higher reaching even 100% in some isolated populations [3]. The proportion of patients devoid of the D-antigen (Rh factor) was substantial (19.37%) compared to both national and global data. About 89–95% donors all over the world are detected as Rh positive. Mason and colleagues detected 37 (4.2%) Rh negatives among 878 Ethiopian soldiers during the Korean War [17]. The proportion of Rh factor negatives ranged from 1–7% in more representative report from Ethiopia [19]. In a recent small-scale study in south-central Ethiopia the proportion of Rh negative subjects ranged from 9–8% [23].

The frequency of the Rh-negative phenotype differs significantly between populations. In Africa and Asia the Rh-negative phenotype is less common. For example, there are reports of a 6% rate of Rh-negatives in Nigeria [24] and only 1% in Madagascar [25]. In various regions of India Rh negativity was found to be 0.6–8.4% [26]. In South East Asia and Far East the D-negative phenotype is even rarer. In China [27, 28], Indonesia [29], and Japan [18] less than 1% of the population is Rh-negative.

On the other hand, Western nations like Britain [30] and United States [31] have Rh factor negativity of 17 and 15%, respectively, which are closer to the findings of this study. A study in one region of Saudi Arabia revealed that 29% of the population was Rh negative [32]. Rh-negative frequencies of about 29% were documented among Basques and in distinct populations living in the High Atlas Range of Morocco [25], which have the highest reported prevalence of Rh-negative phenotypes apart from that from Saudi Arabia above.

The higher frequency of group ‘O’ than non-‘O’ phenotypes among the Nilotic natives in this study agrees with the hypothesis that in malaria endemic areas type ‘O’ is dominant. The Nilotic people are natives to the study area which is among the highest year-round malaria transmission areas in Ethiopia. It appears that the natives with blood type ‘O’ better survived severe malaria. On the other hand, both Rh-negative and ‘O’ phenotypes might have encouraged malaria transmission in the area as asymptomatic carriers prevailed probably because of their resistance to malarial disease showing the beneficial effects of the phenotypes.

As aforementioned, in Africa Rh negativity is very low (1–3%) with the exception of the Yorubas, Chad or Cameroon areas [29]. Previous reports for Ethiopia showed that the Rh negativity in the country to be three times that of the continental average.

The current data calls for routine screening of pregnant women to avoid the potential risk of erythroblastosis fetalis in the study area. In fact both women and men are required to be tested before having a child. But this is rarely practiced in Ethiopia and the current finding would be helpful in creating awareness. Moreover, since Rh-negative blood group is rare in many populations it is scarcely available in blood banks. The finding shows that Rh-negative group is more common than expected among certain groups and such individuals may be approached, encouraged and convinced to donate blood to make this blood group more available in blood banks for the needy and better save life.

## Conclusions

The study population had a comparable ABO blood group distribution to the global distribution. Phenotypes ‘O’ and ‘A’ were dominant among the Nilotic natives and ‘highlanders’ in Gambella Town, respectively. The finding demonstrated that Rh-negativity in some parts of Ethiopia is much higher compared to previous reports and strengthens the observation that over half the Ethiopian gene pool is related to Semites, Berbers and Europeans and not to sub-Saharans. More comprehensive studies in Gambella as well as other parts of Ethiopia are required to determine the ABO-Rh(D) blood group frequencies nationwide.

## Abbreviations

Rh: rhesus factor
