# Correction to: High rhesus (Rh(D)) negative frequency and ethnic-group based ABO blood group distribution in Ethiopia

**DOI:** 10.1186/s13104-018-3281-1

**Published:** 2018-03-14

**Authors:** Lemu Golassa, Arega Tsegaye, Berhanu Erko, Hassen Mamo

**Affiliations:** 1 Aklilu Lemma Institute of Pathobiology, Addis Ababa University, P.O. Box 1176, Addis Ababa, Ethiopia; 2 Jimma University, P.O. Box 378, Jimma, Ethiopia; 3 Department of Microbial, Cellular and Molecular Biology, College of Natural Sciences, Addis Ababa University, P.O. Box 1176, Addis Ababa, Ethiopia

## Correction to: BMC Res Notes (2017) 10:330 10.1186/s13104-017-2644-3

Following publication of the original article [1] the authors reported that the information in Ref. [32] had been misquoted. The Rh factor in one region in Saudi Arabia is 8.8%, not 29% as stated.
